# The Book World of Medicine and Science

**Published:** 1905-11-04

**Authors:** 


### THE BOOK WORLD OF MEDICINE AND SCIENCE.

Indigestion: The Diagnosis and Treatment of the Functional Derangements of the Stomach. By George Herschell, M.D., Lond. (London: Henry J. Glaisher. 1905. Pp. 293. Price 5s.)

This, the third edition, has been almost entirely rewritten, and is intended as an aid to the diagnosis and treatment of the various forms of indigestion so frequently met with in the daily routine of private practice. The treatment of atony of the stomach and the gastric neuroses is very fully discussed, and the author gives details of many interesting cases. The usefulness of the book is much increased by the addition of a section dealing with the cooking of food, a matter which, if carefully detailed, is likely to enhance the patient's confidence in his doctor's ability. The style of the book is good and the price moderate.

A Healthy Home and How to Keep It. By Florence Stacpoole. (London: Wells, Gardner, Darton and Co. In two parts. Price 4d. each part.)

It is often a difficult affair to criticise a book from the standpoint of the particular class of readers for whom it is intended. This is a case in point, for the author evidently has intended this little manual for the most ignorant classes alone. Some of the advice which is given, notably that with regard to the preservation and treatment of bad meat is astonishing. "Musty meat," we are told, "should be washed well with vinegar before cooking. It will then be as good as ever." Decomposing meat is said to be rendered quite wholesome by giving it a bath in a solution of potassium permanganate," but, the author adds, "never do anything with tainted fish but burn or bury it." The author falls into a common and most harmful fallacy with regard to the souring of milk. In view of such lapses from scientific rectitude as these we must regard the book as one which contains very dangerous advice, and on that account we cannot commend it.

